# Tuberculosis active case-finding interventions and approaches for prisoners in sub-Saharan Africa: a systematic scoping review

**DOI:** 10.1186/s12879-020-05283-1

**Published:** 2020-08-05

**Authors:** Desmond Kuupiel, Portia Vezi, Vitalis Bawontuo, Ernest Osei, Tivani P. Mashamba-Thompson

**Affiliations:** 1 Department of Public Health Medicine, School of Nursing and Public Health, University of KwaZulu-Natal, 2nd Floor George Campbell Building, Durban, 4001 South Africa; 2 Research for Sustainable Development Consult, Sunyani, Ghana; 3 Faculty of Health and Allied Sciences, Catholic University College of Ghana, Fiapre, Sunyani, Ghana; 4 Department of Public Health, Faculty of Health Sciences, University of Limpopo, Polokwane, South Africa

**Keywords:** Tuberculosis, Active case-finding, Approaches, Prisoners, Sub-Sahara Africa

## Abstract

**Background:**

In sub-Saharan Africa (SSA), most prisons are overcrowded with poor ventilation and put prisoners disproportionally at risk of exposure to *Mycobacterium tuberculosis* (TB) and developing TB infection but are mostly missed due to poor access to healthcare. Active case-finding (ACF) of TB in prisons facilitates early diagnosis and treatment of inmates and prevent the spread. We explored literature and described evidence on TB ACF interventions and approaches for prisoners in SSA prisons.

**Methods:**

Guided by the Arksey and O’Malley framework, we searched PubMed, Google Scholar, SCOPUS, Academic search complete, CINAHL and MEDLINE with full text via EBSCOhost for articles on prisoners and ACF from 2000 to May 2019 with no language restriction. Two investigators independently screened the articles at the abstract and full-text stages in parallel guided by the eligibility criteria as well as performed the methodological quality appraisal of the included studies using the latest mixed-method appraisal tool. We extracted all relevant data, organized them into themes and sub-themes, and presented a narrative summary of the results.

**Results:**

Of the 391 eligible articles found, 31 met the inclusion criteria. All 31 articles were published between 2006 and 2019 with the highest six (19.4%) in 2015. We found evidence in 11 countries. That is, Burkina Faso, Cameroon, Coˆte d’Ivoire, the Democratic Republic of the Congo, Ethiopia, Ghana, Malawi, Nigeria, South Africa, Uganda, and Zambia with most 41.9% (13/31) recorded in Ethiopia. These intervention studies were conducted in 134 prisons between 2001 and 2018 using either a single or combination of mass, facility-led, entry, peer educators for routine screening, and exit ACF approaches. The majority (74%) of the studies utilized only a mass screening approach. The most (68%) reported study outcome was smear-positive TB cases only (68%). We found no evidence in 16 SSA countries although they are classified among the three high-burden country lists for TB TB/HIV and Multidrug resistant-TB group.

**Conclusion:**

Our review highlights a dearth of evidence on TB ACF interventions in most SSA countries prisons. Hence, there is the need to scaling-up ACF interventions in SSA prisons, particularly countries included in the three high-burden country lists for TB, TB/HIV, and MDR-TB.

## Background

Tuberculosis (TB) is a major global health problem. TB affects about 30% of the world’s population and is the topmost cause of mortality from a sole infectious agent accounting for more than 1.3 million deaths yearly [1–4]. It is estimated that appropriately 94% of all TB infections and deaths occur in low- and-middle-income countries, including Sub-Saharan Africa (SSA) [4]. Although more than 10 million estimated TB new cases occurred in 2017 worldwide, almost a third of these were missing cases (either not notified or undiagnosed or unreached by the health system) [3–7]. However, the goal of the World Health Organization (WHO) End TB Global Strategy is to detect an estimated 90% TB cases particularly, among TB key populations considered to be most at risk of TB infection and yet have limited access to quality healthcare services for TB [1, 8, 9]. Inmates or prisoners are classified among TB key populations with a high risk of exposure to *Mycobacterium tuberculosis* and developing TB infection but are mostly missed owning to several known reasons [5, 10–12].

Research has demonstrated higher risks of acquiring and developing TB infection in prison settings compared to the general population [13–16]. This is commonly due to overcrowding, poor ventilation, poor nutrition, comorbid illnesses such as HIV, and poor access to TB services [13, 14, 17], especially in SSA countries prisons. In 2016 for instance, a review involving 24 SSA countries prisons showed TB prevalence ranging from 0.4 to 16.3% [18]. In the same year, Dolan et al. also revealed regional variations of TB prevalence in SSA countries prisons estimated at 5.3% in East and Southern Africa and 2.9% in West and Central Africa [18]. Notwithstanding this, disparities in accessing TB diagnosis and treatment services still exist in SSA countries especially, for the prison population contributing to a lack of knowledge and late detection of TB cases in prisons. To this end, the WHO recommends the integration of TB services in prisons with national TB programs [18, 19]. The WHO further recommends active case finding (ACF) of TB in prisons to facilitate early diagnosis and treatment of inmates with TB infection as well as prevent the spread of the disease [18].

In general, ACF is a systematic identification of people with suspected active TB, in a predetermined target group or population, using tests, examinations, or other procedures that can be applied rapidly [20, 21]. TB ACF in prisons may involve approaches such as mass screening, entry screening, routine screening, and exit screening. ACF of TB cases contributes to increasing TB case notification and treatment success rates and reducing mortality [22–26]. Hence, there is the need to sustain active search for TB cases in prisons, particularly in SSA where the burden of TB is still much higher compared to other regions in the world. TB ACF intervention studies alongside political will and commitment are essential towards ending TB among vulnerable and key populations. Studies aiming at identifying research gaps are also crucial for future research to inform TB ACF policies in prisons. Despite this, to date, no study has comprehensive review literature on TB ACF and approaches in SSA prisons to inform policy and reveal research gaps. Therefore, this study systematically explored literature and described the scope of evidence on TB ACF interventions and approaches for prisoners in SSA to address this gap in the literature.

## Methods

This review conforms to the Arksey and O’Malley framework, Levac et al. 2010 recommendations, and the Joanna Briggs Institute 2015 recommendations [27]. A detailed description of the method has been previously reported in the published protocol [28]. This study is part of a larger scoping review; however, the present study focused on evidence of TB ACF interventions for prisoners in SSA countries prisons. We followed the preferred reporting items for systematic and meta-analyses extension for scoping reviews (PRISMA-ScR) checklist to report this study [29] (Supplementary file 1).

### Identifying the research question

The research question for this was: What is the evidence on TB active case-finding intervention in SSA Countries prisons? To determine the eligibility of this research question for the scoping review, the population, concept, and context (PCC) mnemonic [27] was used, as illustrated in Table 1.

**Table 1:** PCC framework for defining the eligibility of the scoping review question

P-Population	Prisoners of all ages: Inmates still in incarceration in prisons
C-Concept	TB active case-finding: the systematic identification of people with a suspected TB infection, in prison population, using tests, examinations or other procedures that can be applied rapidly.
C-Context	Prisons in SSA countries

### Identify relevant studies

We first searched the following six academic databases from 2000 to May 26, 2019: PubMed, Google Scholar, SCOPUS, Academic search complete, CINAHL with full text, and MEDLINE with full text via EBSCOhost in consultation with an experienced librarian, we developed a search strategy using the following combination of keyword for each database. “Prisoners” “prisoners” “prisoner*”, “inmates”, “TB”, “tuberculosis” “tuberculos*” “Koch diseas*”, “mycobacterium”, “active surveillance”, “Watchful Waiting”, “Watchful Waiting*”, “active case finding”, “case finding”, “active search”, and “surveillance”. MeSH terms and Boolean terms (AND/OR) were included in the search. Date, language, and study design limitations were removed during the search (See Supplementary file 2 for the full search strategy in the electronic databases). Secondly, we screened for relevant articles in the reference lists of the included studies.

### Eligibility criteria and study selection

Inclusion criteria were as follows: articles presenting evidence from SSA countries as defined by the WHO; studies involving prisoners of all ages; quantitative descriptive studies or interventional or implementation studies reporting evidence on ACF of TB; and papers published in English from January 2000 to May 2019. This study’s exclusion criteria was articles reporting tuberculosis ACF among the general population; studies conducted among prisoners but did not include tuberculosis ACF; and studies conducted in other low-and-middle-income countries as well as high-income countries that are not classified among the WHO Africa Region; reviews; qualitative studies; and conference proceedings.

To reduce bias, the articles were independently screened in three stages. That is titles screening (DK), abstract screening, and screening of full-text articles by two investigators (DK and PV) using the eligibility criteria as a guide after deletion of duplicates from the endnotes library specially created for this review. We utilized the University of KwaZulu-Natal library services as well as sent emails to authors requesting those full texts that were not accessible online for screening. At the title and abstract screening stages, disagreements were resolved by the reviews (DK and PV) through discussions until a conclusion was reached. A third reviewer (EO) resolved the discrepancies following full-text screening.

### Charting the data

Prior to full data extraction, two reviewers (DK and EO) piloted the data extraction form developed in google forms using ten randomly sampled included studies. Then DK and EO compared the data extracted for consistency and accuracy. Then, we addressed discrepancies and the data extraction form amended. Using the amended data extraction form, DK and EO once again independently extracted data for another ten randomly studies and once more cross-checked for consistency and accuracy. Following this, DK extracted data from the remaining included studies using the amended data extraction form. Five core categories of data were abstracted: Details of the authors and publication year, methodological characteristics, characteristics of the study sample, TB ACF intervention and strategies, and study outcomes.

### Collating, summarizing, and results

Following the extracted of all the relevant data from the included studies, we conducted a thematic analysis. The extracted data were organized into themes and sub-themes, and the summary of the results presented narratively. Emerging themes were also reported.

### Quality appraisal

The quality of each included study was appraised using a modified version of the 2018 mixed-method quality appraisal tool (MMAT) [30]. The MMAT tool has two screening questions and a set of five questions for each of study design included (Randomised control trail, Non-randomised control, and quantitative descriptive studies) as shown in supplementary file 3. Two reviewers (DK and EO) performed the quality appraisal and scored the included studies independently using the two screening questions and a set of five questions each for the included randomized controlled trial, non-randomized study, and quantitative descriptive studies as prescribed by the MMAT. A total percentage score was calculated by adding all the items rated, divided by seven, and multiply by a hundred. Studies that scored less than 50% were interpreted as low quality whilst studies that scored from 51 to 70% were interpreted as average quality. Finally, a study was interpreted to be of high methodological quality if it scored greater than 70% [28].

## Results

**Fig. 1:**
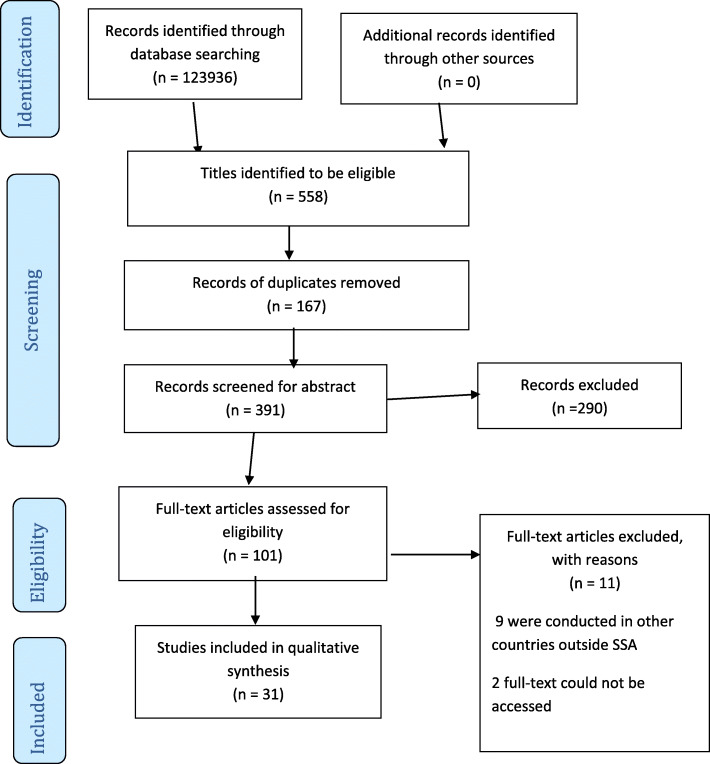
PRISMA 2009 Flow Diagram

Of the 123,936 studies yielded by this scoping review from the database search, 558 articles met the eligibility criteria following title screening. Prior to the abstract screening of the 558 articles, 167 duplicates were identified and removed further reducing the number of potentially relevant articles to 391 for abstract screening (Fig. 1). Based on the inclusion and exclusion criteria of this study, 349 articles were additionally removed following the abstract screening. Of the 42 potentially relevant articles independently screened at the full-text screening stage, only 31 articles were identified to be eligible and were included for data extraction. Nine (9) of the excluded studies at the full-text stage were conducted in other jurisdictions outside the SSA region, and the full-text of two articles could not be accessed since the authors did not respond to our email request.

### Characteristics of the included studies

**Fig. 2:**
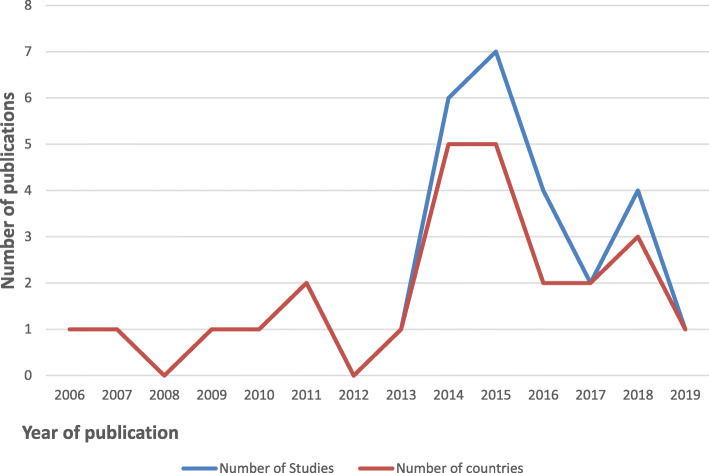
A line graph comparing the number of publications and countries per year

All 31 studies included in this review reported evidence of TB ACF interventions in eleven countries prisons (Burkina Faso, Cameroon, Coˆte d’Ivoire, Democratic Republic of the Congo (DRC), Ethiopia, Ghana, Malawi, Nigeria, South Africa, Uganda, and Zambia). The majority, 41.9% (13/31) of the ACF intervention studies were conducted in Ethiopia, and one study each in Ghana, Burkina Faso, Cameroon, Malawi, and Coˆte d’Ivoire. There was no evidence of TB ACF found for 43 (79.6%) of the total 54 African countries, although WHO has classified 16 of them among countries in the three high-burden country lists for TB TB/HIV and MDR-TB [31]. All the included studies were published from 2006 to 2019. Approximately 22.6% (7/31) of the studies were published in 2015 from five countries whilst 6 (19.4%) were published in 2006, 2007, 2009, 2010, 2013, and 2019 from six countries. There was no publication in 2008 and 2012 (Fig. 2).

However, the studies included showed the ACF interventions were implemented between 2001 and 2018 with the highest number in 2013 and 2015 (*n* = 5 each). This finding reveals a downward trend of TB ACF intervention between 2015 and 2018. Of the total 134 prisons where TB ACF intervention studies were conducted, the majority (44) were in Ethiopia, Malawi (*n* = 18), Uganda (*n* = 17), Zambia (*n* = 17), South Africa (*n* = 5), Nigeria (*n* = 3), Coˆte d’Ivoire (*n* = 3), DRC (*n* = 2), Ghana (*n* = 1), Cameroon (*n* = 1), and Burkina Faso (*n* = 1). This finding demonstrates limited TB ACF intervention studies in SSA countries prisons. Twenty-eight (90.3%) of the included studies were cross-sectional studies [32–59] two cohort studies [60, 61] and one cluster-randomized trial [62]. This finding reveals limited randomized control trials to determine the feasibility and effectiveness of other innovative TB ACF finding approaches such as peer-led in prisons (Table 2).

**Table 2:** Characteristics of the included studies

Author and publication year	Country	Study design	Name/ number of prison(s)	Intervention year	Laboratory diagnostic method	Outcome reported
Abebe et al., 2011 [32]	Ethiopia	Cross-sectional study	Jijiga, Dire Dawa, and Harar	2008	Direct smear microscopy and culture	Active TB
Adane et al., 2016 [33]	Ethiopia	Cross-sectional study	Axum, Mychew, Adwa, Adigrat, Shire, Humera, Alamata, Mekelle, and Wukro	2014	Acid fast bacilli microscopy and culture	Active TB
Adane et al., 2019 [62]	Ethiopia	Cluster-randomized trial	Mekelle, Shire, Adawa, Abi Addi, Humera, Adigrat, Maichew, Alamata, Wukro, Axum, Dessie, Woldia, Fenote Selam, Debre Markos, Debre Tabor, and Bahir Dar.	2017	Clinical re-evaluation based on physician’s judgment, Xpert MTB/RIF assay with new sample, Culture and drug susceptibility testing, and chest X-ray	Active TB
Addis et al., 2015 [34]	Ethiopia	Cross-sectional study	Gondar Prison	2008	Acid fast staining technique	Active TB
Adesokan et al., 2014 [35]	Nigeria	Cross-sectional study	Agodi prison	2013	Löwenstein-Jensen slopes with pyruvate and/or glycerol	Active TB
Agajie et al., 2018 [36]	Ethiopia	Cross-sectional study	Metekel, Assosa, and Inkamash	2018	Gene expert	Active TB
Ali et al., 2015 [37]	Ethiopia	Cross-sectional study	Ambo, Asebeteferi, Asella, Bonga, Dilla, Harar, Jimma, Mizan, Nekemte, Shashemene, Sodo, Wolkite, and Yabelo	2013	Smear microscopy and solid culture	Active TB
Banda et al., 2009 [38]	Malawi	Cross-sectional study	Zomba Central, Chichiri, Maula, Chitipa, Karonga, Mzuzu, Ntcheu, Mwanza, Mikuyu, Nkhota-kota, Mulanje, Nkhata-bay, Kasungu, Chikwawa, Mzimba, Mpsyupsyu, Mangochi, and Domasi	2005	Sputum smear microscopy	Active TB
Biadglegne et al., 2014 [39]	Ethiopia	Cross-sectional study	Debrebrihan, Dessie, Woldia, Gondar, Debiretab, Bahir Dar, Fenoteselam, and Debremarkos	2013	Acid fast bacilli microscopy	Active TB
Chigbu et al., 2010 [60]	Nigeria	Cohort study	Aba Federal Prison	2007	Sputum-smear microscopy and sputum culture and tuberculin skin testing	Active TB and LTBI
Ekundayo et al., 2015 [41]	Nigeria	Cross-sectional study	Aba Federal Prison	2015	Sputum smear microscopy	Active TB
Diendere et al., 2011 [40]	Burkina Faso	Cross-sectional study	Ouagadougou prison	2009	BAAR detection was performed by direct examination of sputum	Active TB
Fuge et al., 2016 [42]	Ethiopia	Cross-sectional study	Hadiya Zone prison	2013	Compound light microscopy	Active TB
Gebrecherkos et al., 2016 [43]	Ethiopia	Cross-sectional study	Debark, Dabat, Chilga, and Gondar towns of North Gondar zone	2015	Acid fast bacilli and GeneXpert MTB/RIF assay	Active TB
Beza et al., 2017 [44]	Ethiopia	Cross-sectional study	Motta, Bichena, and Debre Markos	2016	Gene Xpert MTB/RIF	Active TB
Habeenzu et al., 2007 [45]	Zambia	Cross-sectional study	Central Lusaka, Kamwala Remand Lusaka, Mwenbeshi Open Air, Mazabuka District, Monze Open Air Choma State, Kalamo District, Livingstone Central, Mukobeko Maximum Kabwe, Mukobeko Medium Kabwe, Mpima Kabwe, Kansenshi Ndola, and Kanfinsa Kitwe	2001	Cultured for *Mycobacterium tuberculosis*. Antimicrobial resistance testing was performed by the resistance ratio method	Active TB
Harris et al., 2014 [61]	Zambia	Cohort study	Lusaka Central Prison	2011	Lowenstein-Jensen medium, liquid Mycobacterial Growth Indicator Tube 960 System, BD Microbiology Systems, and culture. Species identification and drug susceptibility testing performed with Genotype MTBDRplus	Active TB
Henostroza et al., 2013 [46]	Zambia	Cross-sectional study	Lusaka Central Prison	2011	Digital X-ray/sputum smear microscopy, LED fluorescence, Lowenstein-Jensen Medium) culture.	Active TB
Kalonji et al., 2016 [47]	Democratic Republic of the Congo	Cross-sectional study	Mbuji-Mayi Central Prison	2015	Clinical examination and bacteriological tests	Active TB
Karamagi et al., 2018 [48]	Uganda	Cross-sectional study	16 central and farm prisons (Names not specified)	2017	Combined quality improvement with facility-led active case finding (QI-ACF)	Active TB
Kayomo et al., 2018 [49]	Democratic Republic of the Congo	Cross-sectional study	Mbuji-Mayi Central Prison	2015	Xpert MTB/RIF assay	Active TB
Kwabla et al., 2015 [50]	Ghana	Cross-sectional study	Ho Prison	2014	Sputum smear microscopy	Active TB
Maggard et al., 2015 [51]	Zambia	Cross-sectional study	Lusaka Central Prison, Livingstone Central Prison, Kabwe Prison Complex,	2011	Fluorescence microscopy, cultures, X-rays and physical examinations.	Active TB
Merid et al., 2018 [52]	Ethiopia	Cross-sectional study	Hawassa prison	2016	Acid-fast bacilli, smear microscopy and molecular diagnostic testing (Xpert® MTB/RIF)	Active TB
Noeske et al., 2006 [53]	Cameroon	Cross-sectional study	New Bell Central Prison of Douala	2004	Smear microscopy and/or culture	Active TB
Owokuhaisa et al., 2014 [54]	Uganda	Cross-sectional study	Mbarara Central prison located in Kiswahili cell Mbarara	2012	Ziehl-Neelsen technique	Active TB
Seri et al., 2017 [55]	Coˆte d’Ivoire	Cross-sectional study	three buildings of MACA: the men’s longterm detention building, the women’s detention building, and the infirmary inpatients ward	2015	Systematic direct smear microscopy, culture and chest X-ray)	Active TB
Telisinghe et al., 2014 [56]	South Africa	Cross-sectional study	Male’s prison block housing sentenced prisoners	2010	Chest radiograph and two spot sputum specimens for microscopy and culture.	Active TB
Winsa et al., 2015 [57]	Ethiopia	Cross-sectional study	Bedele woreda prison	2012	Ziehl-Neelsen staining technique	Active TB
Zerdo et al., 2014 [58]	Ethiopia	Cross-sectional study	The three prisons of Gamo Goffa Zone	2012	Sputum smear microscopy and culture on Lowenstein-Jensen media	Active TB
Zishiri et al., 2015 [59]	South Africa	Cross-sectional study	4 facilities (2 large and 2 smaller)	2013	GeneXpert MTB/Rif)	Active TB

### Quality of evidence

All the 31 articles included in this study underwent methodological quality appraisal using the latest MMAT version. All 31 articles scored between 57 and 86%. The majority (45.2%) of the included articles scored 71.4% (14/31) [36, 39, 40, 42, 45, 46, 49, 50, 53–56, 60, 61] and 10 (32.2%) scored 57.1% [35, 37, 38, 41, 43, 44, 47, 48, 52, 57]. The remaining seven included articles scored the highest (85.7%) [32–34, 51, 58, 59, 62].

### Study findings

#### ACF approaches utilized by included studies

**Fig. 3:**
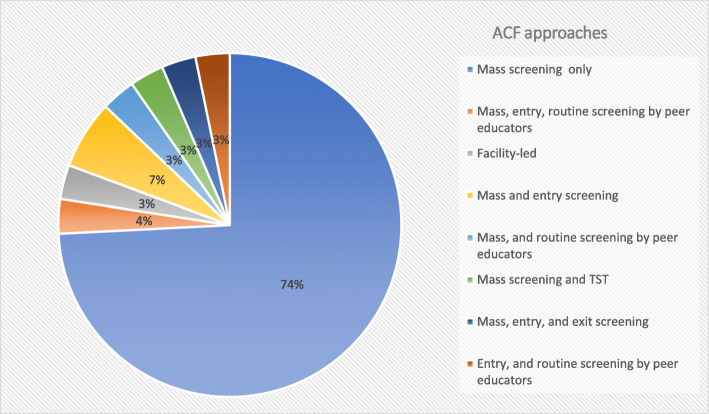
Summarized TB ACF approaches used by the included studies

Of the 31 included studies 23 (74%) used only mass screening strategy [32–45, 47, 49, 50, 52–55, 57, 58], and two studies used both mass and entry screening [56, 61]. One study each used the following strategies: Mass screening and routine screening by peer educators [62], mass screening and tuberculin skin testing [60], facility-led ACF [48], mass screening of inmates, community-based screening of adjacent encampments, and comprehensive entry and routine using inmates as peer educators [51], entry screening and screening of newly admitted prisoners, and use of inmates as peer educators [59], and mass screening of all inmates entering, residing, and exiting the prison, as well as in the surrounding community [46]. Figure 3 summarizes the ACF approaches used by the included studies. This finding shows limited studies utilizing facility-led, peer-led, as well as entry, routine, and exit TB ACF approaches in SSA prisons.

#### ACF interventions and outcomes

**Fig. 4:**
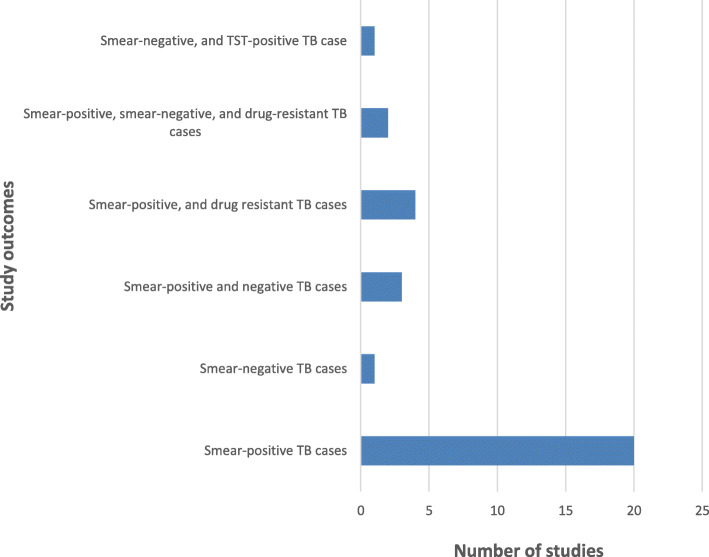
Distribution of TB ACF interventions outcomes reported by the included studies

All 31 studies included in this scoping review reported on active TB infections [32–62]. However, one study additionally presented evidence on latent TB infection (LTBI) [60]. A majority (64.5%) of the included studies reported on only smear-positive PTB cases, and one (3%) each reported on only smear-negative PTB cases, and smear-negative PTB and LTBI (Fig. 4).

#### Smear-positive PTB cases

Out of the 31 included studies involving TB ACF, 30 (97.8%) reported evidence on sputum smear-positive PTB cases [32–38, 40–62]. In Ethiopia, Abebe et al. study in Jijiga, Dire Dawa, and Harar prisons identified 8.9% (33/371) prisoners with confirmed smear- or culture-positive PTB [32]. Adane et al. study in reports in 2016 involving nine prisons confirmed a 4% (32/809) undiagnosed TB cases [33], and again in 2019, confirmed almost 7% (75/1124) of new TB cases from their cluster-randomized trial aimed to improve TB case detection in Ethiopia of which 5.4% (4/75) were smear-positive [62]. In a cross-sectional study involving 384 prisoners, Addis et al. reported the prevalence of smear-positive PTB cases with a cough duration of more than 2 weeks as 8.59% among Ethiopian prisoners [34]. In Agajie et al. a total of eight (9.5%) new PTB cases were found among 84 prisoners with a cough duration of more than 2 weeks in Ethiopia [36]. In Ali et al. 2015 study, nearly 5% (765) TB were identified as TB suspects from 15,495 prisoners and 2.8% (20) new culture-confirmed TB cases were identified in Ethiopia [37]. In the Hadiya Zone Prison of Ethiopia, Fuge et al. reported 349.2 per 100,000 populations point prevalence of smear-positive PTB cases in their study involving 164 prisoners [42]. Gebrecherkos et al. and Beza et al. also identified 5.3% (15/282) and 3.4% (9/265) smear-positive PTB cases in their respective studies in Ethiopia [43, 44]. Similarly, Merid et al. identified 372 suspected TB cases among 2068 prisoners of which eight (2%) were AFB sputum smear-positive and 31 (8%) were Xpert-positive [52]. Moreover, Winsa et al. and Zerdo et al. found 21.9% (43/196) and 19.4% (24/124) in their respective cross-sectional studies conducted in Ethiopia [57, 58].

In Nigeria, Adesokan et al. study in Nigeria aimed to determine the prevalence of TB in Agodi prison reported two new PTB cases among 164 prisoners [35]. Chigbu et al. identified 5.42% (91/1680 prisoners with infections owed to Mycobacterium, of which 3.3% (3/91) were sputum-smear- and culture-positive [60]. Ekundayo et al. study aimed to evaluate the contribution of ACF to TB control in Aba Federal Prison of Nigeria clinically screened 449 inmates and identified 21.15% TB cases out of the 52 eligible inmates tested for sputum smear microscopy [41].

In Zambia, four (12.9%) studies presented evidence of TB ACF among inmates. Habeenzu and colleagues’ study in 2007 involving 1080 inmates recruited from twelve prisons in Zambia yielded 168 (15.6%) inmates with smear-positive disease [45]. Harris et al. 2014 cohort study in the Lusaka Central Prisons involving 1487 inmates reported 62 (4.2%) culture-confirmed TB of which eleven (18%) were smear-positive PTB cases [61]. Henostroza et al. undertook mass screening of all inmates entering, residing, and exiting the prison, as well as in the surrounding community of the Lusaka Central Prison and diagnosed new TB cases in 7.6% (176/2323) of which 50% were bacteriologically confirmed [46]. ACF interventions at entry and exit yielded 4.6 and 5.3% confirmed TB cases respectively, and 25% (*n* = 22/88) smear-positive of the bacteriologically confirmed TB cases [46]. Similarly, Maggard and colleagues’ study aimed to improve the Zambia Prisons Service’s implementation of TB screening and HIV testing undertook a mass screening of inmates and community-based screening of those residing in encampments adjacent to prisons as well as routine screening of inmate using peer educators [51]. Their intervention identified an additional 409 new TB cases of which 160 were bacteriologically positive either by fluorescence microscopy or culture or both [51].

Of the 31 studies reporting evidence of TB ACF interventions, two each were conducted in the Democratic Republic of the Congo (DRC), Uganda, and South Africa. In the DRC, Kalonji et al. 2016 cross-sectional study aimed at estimating TB prevalence in the population of Mbuji-Mayi Central Prison via mass screening approach, confirmed 130 prisoners with TB, representing 17.7% TB prevalence [47]. Kayomo et al. cross-sectional study aimed to report the outcomes of the outbreak of TB and Multidrug-Resistant TB in Mbuji-Mayi Central Prison further confirmed 170 TB cases out of 475 symptomatic TB cases [49]. In Uganda, Karamagi and colleagues combined quality improvement with facility-led active case finding confirmed 2.3% (34/1494) TB cases from 16 central and farm prisons in Northern Uganda [48]. Owokuhaisa et al. ACF intervention in Mbarara Central prison of Uganda involving 248 prisoners confirmed five new TB cases [54]. In South Africa, Telisinghe and colleagues utilized entry and mass screening in a study aimed to determine the prevalence of active undiagnosed PTB in the prison block housing sentenced male prisoners and reported 3.5% (34/968) prevalence of undiagnosed TB cases of which six (17.6%) were culture positive for M.TB [56]. Zishiri et al. in a program evaluation to describe reach, effectiveness, adoption, implementation, and maintenance of ACF interventions in 4 correctional facilities in South Africa screened 7426 prisoners and confirmed 201 (2.7%) PTB cases [56].

Of the 31 included studies, one each reported evidence of TB ACF interventions among prisoners in Malawi, Burkina Faso, Ghana, Cameroon, and Coˆte d’Ivoire. Banda et al. study aimed to determine the period prevalence of smear-positive PTB in Malawi undertook a mass screening of 7661 inmates from eighteen prisons and confirmed 54 (0.7%) smear-positive PTB cases [38]. In Burkina Faso, Diendere and colleagues’ mass screening intervention of inmates in Ouagadougou prison confirmed 1.3% (4/308) PTB cases [40]. Kwabla and colleagues’ mass screening of 151 prisoners from the Ho prisons of Ghana yielded one smear-positive PTB cases [50]. In Cameroon, Noeske et.al. cross-sectional study involving inmates in New Bell Central Prison of Douala found 60 inmates confirmed sputum smear and/or culture-positive in a series of sputum smear examinations [53]. Seri et al. found 59 (6.2%) prisoners with sputum smear-positive TB out of 943 prisoners screened in their study aimed to estimate the prevalence of PTB among prisoners at the largest prison of Coˆte d’Ivoire following 16 years TB program implementation [55]. Figure three below illustrates the distribution of the countries where the included studies were conducted.

#### Smear-negative TB cases

Eight (25.8%) of the 31 studies included in this review also found smear-negative TB cases from their ACF interventions in prisons. In Ethiopia, Abebe et al. study in Jijiga, Dire Dawa, and Harar prisons diagnosed 5.4% (20/371) prisoners who were smear-negative, culture-positive TB cases [32]. Adane et al. cluster-randomized study aimed to improve TB case detection, and feasibility of interventions based on available resources in Tigray and Amhara prisons of Ethiopia confirmed 61 and 32% smear-negative TB cases in the intervention and control prisons respectively [62]. Biadglegne et al. in their study aimed to obtain initial data on the prevalence of smear-negative cases of TB cases in Ethiopian prisons undertook a mass screening of inmates from eight prisons in Amhara and detected 8% (16/200) total prevalence of smear-negative PTB cases [39]. In Zambia, Henostroza and colleagues undertook mass screening of inmates in the Lusaka central prison and reported that 75% of the 88 bacteriologically confirmed TB cases were smear-negative TB cases [46]. Using mass screening, and routine screening of inmates by peer educators in Lusaka central, Livingstone central, and Kabwe complex prisons, Maggard et al. additionally found 249 of 409 newly diagnosed TB cases to be smear-negative [51]. In Coˆte d’Ivoire, Seri and colleagues’ mass screening of 943 inmates in the men’s long-term detention building, the women’s detention building, and the infirmary in-patients ward and found 3.1% (29) to be smear-negative TB cases [55]. Telisinghe and colleagues’ study in South Africa aimed to determine the prevalence of active undiagnosed PTB in the prison block housing sentenced male prisoners further detected 4% (39/968) smear-negative TB cases [56]. In Nigeria, Chigbu et al. also found that 96.7% (88/91) of the prisoners diagnosed with TB infection were sputum smear-negative [56].

#### Drug-resistant Mycobacterium -TB cases

Six (Ethiopia (*n* = 2), Zambia (*n* = 2), DRC (*n* = 1), and Coˆte d’Ivoire (*n* = 1)) of the thirty-one studies included in this scoping review further detected drug-resistant TB cases from their ACF interventions in the prisons. In Agajie et al. study in Kamash prison of Ethiopia, one of the eight newly diagnosed TB cases was found to be drug-resistant TB [36]. Beza et al., also reported one inmate with rifampicin-resistant TB in their study to determine the prevalence and associated factors of TB in East Gojjam Zone prisons of Northwest Ethiopia [44]. Habeenzu and colleagues’ cross-sectional study aimed to determine the prevalence of undiagnosed TB in 13 Zambian prisons identified 40 and 16 isolates resistance to at least one anti-TB drug, and multidrug-resistance TB respectively [45]. Maggard and colleagues ACF interventions in the Lusaka Central Prison, Livingstone Central Prison, Kabwe Prison Complex additionally detected one of the 160 newly diagnosed inmates had multidrug-resistant TB, whilst four had only isoniazid-resistant TB [51]. In the DRC, Kayomo and colleagues detected an additional 14 inmates with rifampin-resistant TB out of the 199 inmates with confirmed TB [49]. In Coˆte d’Ivoire, Seri et al. using mass screening strategy identified nineteen isolated TB strains of which ten were drug-resistant together with seven multi-resistance TB cases [55]. This finding suggests there are limited studies investigating drug-resistant TB in SSA countries prisons.

#### Latent TB infection

This scoping review found that only one (3.2%) out of the 31 included studies presented evidence on latent TB infection. Chigbu et al. cohort study in Nigeria aimed to determine the transmission of MTB within the Aba Federal prison environment identified a total of 69 inmates who had positive tuberculin skin tests [60].

## Discussions

We conducted a scoping review of published studies to map evidence on TB ACF interventions and approaches for prisoners in SSA from January 2000 to May 2019. This study found 31 eligible studies on TB ACF published between 2006 and 2019. The results show that these TB ACF interventions were implemented between 2001 and 2017 in 134 prisons. These studies were conducted in Burkina Faso, Cameroon, Coˆte d’Ivoire, DRC, Ethiopia, Ghana, Malawi, Nigeria, South Africa, Uganda, and Zambia. However, most (41.9%) of the studies were conducted in Ethiopia. This study finding revealed a downward trend of TB ACF intervention between 2015 and 2018. Also, the review demonstrated limited studies utilizing facility-led, peer-led, as well as entry, routine, and exit TB ACF approaches. The review further revealed limited randomized control trials determining the feasibility and effectiveness of recent TB ACF finding approaches such as peer-led/peer education in prisons. Moreover, this review found that most of the outcomes reported by the published studies were smear-positive PTB cases.

**Fig. 5:**
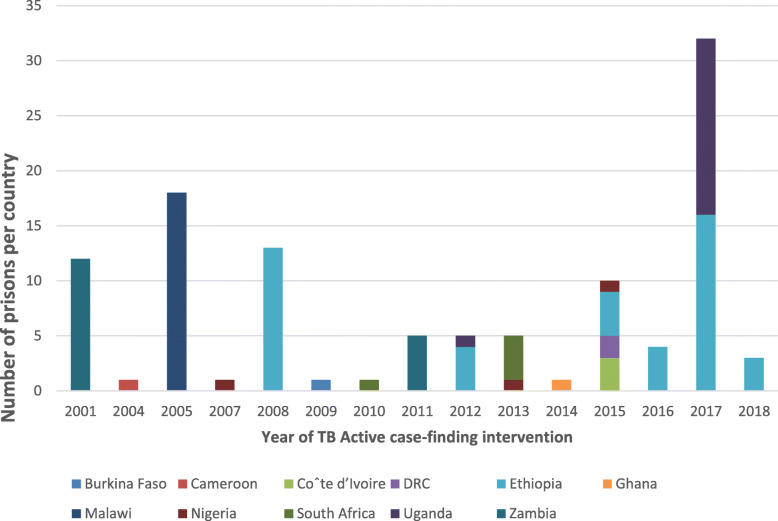
A stacked bar comparing the number of prisons in each country TB ACF interventions were implemented from 2001 to 2018

To the best of our knowledge, this scoping review is the maiden comprehensive review mapping literature on TB ACF interventions and approaches in SSA countries prisons. Nonetheless, our findings correlate with other review studies. We found 31 studies published between 2006 and May 2019. As demonstrated by this current study, the ACF interventions were conducted in a total of 134 prisons (Fig. 5). Data published by the UNAIDS indicates that there are over 900 prisons in SSA [63]. Our study findings, therefore, suggest there is limited research (gap) focusing on TB ACF in SSA prisons. Similarly, Dara et al. in their 2015 systematic review study aimed to describe the challenges of TB control in prisons reported limited research [64]. Tavoschi et.al. in their 2018 systematic review aimed to investigate the available evidence on modalities and effectiveness of ACF interventions in prisons also found vital literature gaps [65].

We also found that more than one-third of the studies were conducted in Ethiopia which supports the 2018 study finding of Merid and colleagues [52]. This potentially will help improve TB case notification rates as well as reduce the TB death rate in Ethiopia. This study found no published articles based on this study’s inclusion criteria reporting evidence on TB ACF in 43 out of the total 54 African countries, although WHO has classified 16 of these countries (Somalia, Angola, Kenya, Mozambique, Central African Republic, Congo, Lesotho, Liberia, Namibia, Tanzania, Botswana, Chad, Eswatini, Serra Leone, Zimbabwe, and Guinea-Bissau) among the three high-burden country lists for TB, TB/HIV, and MDR-TB [31]. But this does not necessarily suggest that TB ACF interventions were not done in those countries within the period. Perhaps, TB ACF interventions findings from those countries exist as grey literature or have been published elsewhere not captured by this study or yet to be published. Nonetheless, several limitations exist with regard to interventions in prisons. These may include ethical issues, access challenges, challenges with monitoring of the intervention, political will and commitment, among others. Despite these, there is a need to initiate or sustain TB ACF in prisons using innovative approaches as well as improve the presence of political will and commitment to end TB in vulnerable and key populations in SSA.

Moreover, our study findings have implications for practice and research. This review found that smear-positive PTB cases were the most reported by the included studies. Notwithstanding, sputum smear-negative, and drug-resistant TB cases were also evinced by this study hence, much more effort is still needed to improve the diagnosis of TB. It is also possible many inmates with TB infection are still undiagnosed in SSA countries. Hence, may affect the attainment of the end TB strategy which aims to end the global TB epidemic, with targets to reduce TB deaths by 95% and to cut new cases by 90% between 2015 and 2035, and to ensure that no family is burdened with catastrophic expenses due to TB infection [66]. We further found few studies utilizing peer-led, entry, routine, and exit TB ACF approaches in prisons. These ACF strategies have been demonstrated to be effective and they ensure continuous screening of new and old inmates for TB infection in prisons [1, 62, 67, 68].

Our findings showed the need for more implementation research focusing on TB ACF in most SSA countries prisons. Whilst there is the need to improve TB ACF research in all SSA countries, we encourage future researchers in those countries we found few or no evidence of TB ACF in prisons and yet they are categorized among the three high-burden country lists for TB, TB/HIV, and MDR-TB [31]. We further recommend more randomized control trials to determine the feasibility and effectiveness of other innovative TB ACF approaches of SSA countries prisons and facilitate their implementation. Moreover, we recommend a meta-analysis to evaluate the impact of TB ACF interventions in SSA prisons for policy decisions.

This scoping review has several limitations. We only searched for articles published in scientific peer-reviewed journals. It is possible relevant country-level information on TB ACF interventions might have been documented in ministries of health reports, annual reports of the sector ministry in-charge of the prisons, and prison policies/guidelines. Similarly, we did obtain information from national and international organizations websites, such as WHO, and the departments of prisons and health. This study was also limited to only SSA countries hence, it cannot be generalized. The date limitation perhaps further excluded studies conducted prior to the year 2000 and after May 2019. Only six search engines in addition to a manual search from the reference list of the included studies were used, which might have resulted in important articles being missed from our search. Lastly, we conducted a scoping review that does not have the same rigorous quality standards compared to systematic review or meta-analysis. Despite these limitations, we believe that our search strategy was comprehensive in reviewing the existing literature on TB ACF interventions and approaches in SSA prisons. We also followed all the steps required of a systematic review except for the registration in PROSPERO. Nonetheless, we published this study protocol in a peer-reviewed journal [28]. By including only peer-reviewed articles, rigorously applying the eligibility criteria for the methodology, and performing methodological quality appraisal we have further maintained quality measures. Moreover, we trust that the published articles included in this review were peer-reviewed and they presented evidence of TB ACF interventions, strategies in SSA prison settings, and the evidence presented by this scoping review is trustworthy.

## Conclusion

Based on this study’s inclusion criteria, the findings suggest there is limited evidence on TB ACF intervention studies in SSA prisons. The available evidence is mainly focused on Ethiopia with few studies conducted in other SSA countries. To facilitate achievement of the end TB strategy by 2030, we recommend a scale-up of TB ACF implementation studies in SSA prisons particularly, in all the countries included in the three high-burden country lists for TB, TB/HIV, and MDR-TB.

## Supplementary information

**Additional file 1: Supplementary file 1:** Preferred Reporting Items for Systematic reviews and Meta-Analyses extension for Scoping Reviews (PRISMA-ScR) Checklist

**Additional file 2: Supplementary file 2:** Electronic databases search results for title screening

**Additional file 3: Supplementary file 3:** Quality appraisal tool

## Data Availability

We have duly cited all articles and data is presented in a form of references.

## Abbreviations

ACF: Active case-finding
TB: Tuberculosis
MDR: Multidrug-resistant
SSA: Sub-saharan Africa
WHO: World health organization
