# Supplementary material for: Tuberculosis active case-finding interventions and approaches for prisoners in sub-Saharan Africa: a systematic scoping review
Source: BMC Infect Dis. 2020 Aug 5;20:570. doi: 10.1186/s12879-020-05283-1 (PMC7405346; doi:10.1186/s12879-020-05283-1)
Supplement: Supplementary file 2 — Additional file 2: Supplementary file 2: Electronic databases search results for title screening [file 12879_2020_5283_MOESM2_ESM.docx]

**Supplementary file 2:** Electronic databases search results for title screening

| **Date** | **Databases** | **Keywords** | **Search results** | **Eligible studies** |
| --- | --- | --- | --- | --- |
| 5/05/2019 | SCOPUS | "active tuberculosis” AND "case finding" | 1282 | 90 |
| 6/05/2019 | Google Scholar | Tuberculosis AND active case-finding AND prisoners | 3800 | 182 |
| 25/05/2019 | PubMed | "tuberculosis"[All Fields] OR "TB"[All Fields] OR "tuberculosis"[MeSH Terms] OR "koch's disease"[All Fields] AND "Tuberculosis"[All Fields] AND "active case finding"[All Fields] OR "surveillance"[All Fields] OR "active searching"[All Fields] OR "case searching"[All Fields] OR "epidemiology"[MeSH Terms] OR "epidemiology"[All Fields] OR "watching"[All Fields] AND "prisoners"[All Fields] OR "Prisoners"[MeSH Terms] OR "inmates"[All Fields] OR "incarcerated"[All Fields] OR "Jail"[All Fields] OR "detainees"[All Fields] | 23957 | 216 |
| 26/05/2019 | Ebscohost (Academic search complete, CINAHL with full text, and MEDLINE with full text | TB [SU Subject Terms] OR Tuberculosis [SU Subject Terms] OR tuberculosis [SU Subject Terms] OR Koch diseas*'' [SU Subject Terms] AND active case finding [SU Subject Terms] OR case finding[SU Subject Terms] OR active surveillance [SU Subject Terms] AND Policy[SU Subject Terms] OR policy [SU Subject Terms] OR guideline [SU Subject Terms] OR strategies [SU Subject Terms] OR Intervention [SU Subject Terms] AND Prisoners [SU Subject Terms] OR prisoners [SU Subject Terms] OR prisoner* | 94,897 | 70 |
| Total |  |  | 123936 | 558 |
